# Green Nanotechnology of Cell Wall Swelling for Nanostructured Transparent Wood of High Optical Performance

**DOI:** 10.1002/smll.202406749

**Published:** 2024-12-17

**Authors:** Hui Chen, Jonas Garemark, Lengwan Li, Mathias Nero, Maximilian Ritter, Ocean Cheung, Tom Willhammar, Ilya Sychugov, Yuanyuan Li, Lars A. Berglund

**Affiliations:** ^1^ Wallenberg Wood Science Center Department of Fibre and Polymer Technology KTH Royal Institute of Technology Stockholm 100 44 Sweden; ^2^ Wood Materials Science Institute for Building Materials ETH Zürich Zürich 8093 Switzerland; ^3^ State Key Laboratory of Organic‐Inorganic Composites School of Materials Science and Engineering Beijing University of Chemical Technology Beijing 100029 China; ^4^ Department of Materials and Environmental Chemistry Stockholm University Stockholm 106 91 Sweden; ^5^ Ångströmlaboratoriet Department of Materials Science and Engineering Uppsala University Uppsala 751 03 Sweden; ^6^ Department of Applied Physics School of Engineering Sciences KTH Royal Institute of Technology Stockholm 114 19 Sweden

**Keywords:** accessibility, cell wall swelling, large‐scale wood delignification, sub‐zero NaOH treatment, transparent wood composites

## Abstract

Transparent wood composites provide new functionalities through active additives distributed at the nanoscale. Scalable nanotechnology includes processing where nanoparticles and molecules are brought into the dense wood cell wall. A novel cell wall swelling step through green chemistry is therefore investigated. Sub‐zero centigrade NaOH treatment provides extensive cell wall swelling. Cell wall accessibility is vastly increased so that chemicals can readily impregnate the nanostructured cell wall. Transparent wood with a thickness of up to 15 mm can therefore be fabricated. The optical transmittance and the attenuation coefficient are improved since the polymer is distributed inside the cell wall as a matrix for the nanoscale cellulose fibrils. The proposed technology paves the way for scalable wood nanoengineering.

## Graphical abstract

Extensive cell wall swelling is achieved through sub‐zero centigrade NaOH treatment, leading to vastly increased cell wall accessibility. High‐performance transparent wood (TW) with a thickness of 15 mm and attenuation coefficient of 0.48 cm^−1^ is then successfully prepared based on the NaOH‐swollen wood. This treatment paves the way for scalable wood nanoengineering and multifunctional wood composites beyond TW.

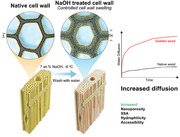

## 1 Introduction

**Figure 1:**
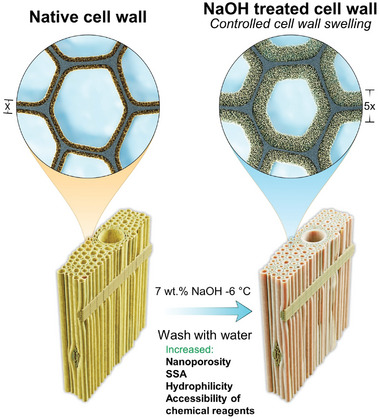
Schematic illustration of wood cell wall swelling process through sub‐zero NaOH treatment.

Multifunctional wood materials based on nanotechnology have the potential to provide new large‐scale wood applications.^[^ ^1^ ^]^ They can combine mechanical properties with, e.g., magnetic,^[^ ^2^ ^]^ optical^[^ ^3^, ^4^, ^5^ ^]^ and/or electrical properties.^[^ ^6^, ^7^ ^]^ Functions for organic electronics can be provided, as exemplified by energy harvesting^[^ ^8^, ^9^, ^10^ ^]^ and storage devices.^[^ ^11^ ^]^ The renewable resource origin of wood is advantageous and its hierarchical structure based on strong cellulose nanofibrils offers opportunities to design complex multiphase materials with good mechanical properties. **Figure** **1** shows a hardwood structure consisting of tubular, hollow fibrous cells organized in the same direction. Typical diameters are 10–30 µm and cell wall thicknesses are in the micrometer range. The cell wall itself is a sophisticated nanocomposite with strong, oriented cellulose nanofibrils embedded in a biopolymer matrix of lignin and hemicelluloses. In the living tree, this matrix is hydrated to ≈30%^[^ ^12^ ^]^ and is therefore designed for this state.

Polymer‐filled transparent wood (TW) composites are attractive since there are not many transparent materials available for engineering applications. Such materials were first prepared for wood anatomy^[^ ^13^ ^]^ studies and then considered for engineering applications.^[^ ^3^, ^5^ ^]^ The first preparation step is delignification or bleaching followed by monomer impregnation; the refractive index of the polymer phase matches the cellulosic wood substrate.^[^ ^3^, ^14^ ^]^ In high‐quality TWs, the polymer is present inside the wood cell wall and distributed at the nanoscale.^[^ ^15^, ^16^ ^]^ The air‐dried cell wall is dense with low porosity and permeability, which is an obstacle to monomer impregnation. Vinyl monomers for thermoplastics are often difficult to bring into the cell wall, due to low solubility in wood tissue. If the cell wall is in a swollen state, this facilitates monomer impregnation.^[^ ^17^ ^]^ Furuno and Goto successfully used a solution of styrene in methanol for cell wall impregnation by diffusion.^[^ ^18^ ^]^ Moustafa used pyridine as a swelling agent to make polymer‐filled wood composites using methyl methacrylate (MMA) and other acrylics,^[^ ^19^ ^]^ but pyridine is toxic. More recently, Cabane et al. did polymer grafting using wood substrates swollen by dimethylformamide (DMF).^[^ ^20^ ^]^

Delignification can remove some of the lignin and hemicelluloses and generate more sub‐micrometer space around cellulose nanofibrils in the cell wall. After delignification, Li et al. reported increased porosity for pores in the diameter region of 4.8 nm.^[^ ^3^ ^]^ Scallan investigated the influence of water on the swelling of delignified pulp fiber cell walls. The specific surface area (SSA) and porosity of the fiber cell wall were increased as water entered.^[^ ^21^ ^]^ In the context of composites, Yano prepared wood/PF composites by first delignifying the wood substrate and then impregnating it with an aqueous, swelling phenol‐formaldehyde solution followed by drying, compression, and curing.^[^ ^22^ ^]^ The material reached high modulus and bending strength. Olsén and colleagues in our lab induced swelling of delignified wood pulp fibers in acetic acid to facilitate polymer grafting inside the cell wall.^[^ ^23^ ^]^ Ansari et al. used delignified chemical wood pulp fibers swollen in methanol for composites, and epoxy reactants successfully diffused into the fiber cell wall.^[^ ^24^ ^]^

For thin wood veneer substrates, delignification is straightforward but thick wood structures are much more difficult to delignify. Strong through‐thickness gradients are common for “degree of delignification”; the outer regions turn into wood pulp while the center is untreated. This is an obstacle for thick multifunctional composites such as polymer‐filled TW, and typical TW thicknesses are 3 mm or below.^[^ ^25^ ^]^ In the current process, wood is often delignified and subjected to solvent exchange. With this procedure, MMA has been successfully brought into the cell wall^[^ ^3^ ^]^ or thiol‐enes^[^ ^16^ ^]^ and even epoxy.^[^ ^26^ ^]^ If cell wall swelling is increased even further, it may be possible to improve the nanoscale distribution of the infiltrated polymer.

Aqueous NaOH is a candidate as a green swelling agent for wood substrates. NaOH is low‐cost, non‐toxic, and non‐hazardous at low concentrations. It may be recycled at an industrial scale, as in existing pulping processes,^[^ ^27^ ^]^ but has not been widely explored for wood nanotechnology. Islam et al. did wood pretreatment at room temperature in 5% NaOH solution before filling with a MMA/styrene monomer mixture followed by polymerization.^[^ ^28^ ^]^ The pretreatment resulted in improved mechanical properties, although mechanisms were not discussed. At room temperature, aqueous NaOH has also been used for cold caustic extraction of hemicelluloses from sulfite pulp to facilitate regenerated cellulose textile fiber processing.^[^ ^29^ ^]^ Zhao et al. did 7% aqueous NaOH pretreatment of wood fiber bundles at −15 °C and at 23 °C which facilitated enzymatic cellulose hydrolysis.^[^ ^30^ ^]^ Again, there was no discussion of mechanisms at the scale of the cell wall.

Here, a scalable wood nanotechnology method is developed using aqueous NaOH pretreatment at sub‐zero temperature. Increased cell wall swelling beyond the water‐swollen native state was observed. We investigate if delignification of thick wood substrates (up to 15 mm) is possible. We further investigate transparent biocomposites, where well‐dispersed cellulose fibrils at the nanoscale are important for optical performance. Total transmittance (photons transmitted compared with incoming light) is improved even in thick composites since the polymer is well mixed with nanoscale cellulose fibrils inside the cell wall.

## 2 Results and Discussion

Wood cell wall swelling is key to truly nanostructured and more homogeneous composites from wood substrates. Here, aqueous NaOH (aq. NaOH) at sub‐zero temperature is for the first time used for this purpose. It was inspired by X‐ray diffraction (XRD) data for low‐temperature aq. NaOH swelling of ramie cellulose reported by Sobue et al.^[^ ^31^ ^]^ In addition, Yamashiki et al. have investigated conditions for cellulose dissolution by aq. NaOH.^[^ ^32^ ^]^ We previously reported an attempt to dissolve and precipitate cellulose fibrils in wood substrates using aq. NaOH at sub‐zero temperature.^[^ ^33^ ^]^ Inspired by this, we here investigate NaOH pretreatment further (Figure 1) and prepare thick TW composites.

### 2.1 Cell Wall Morphology and Composition Change

**Figure 2:**
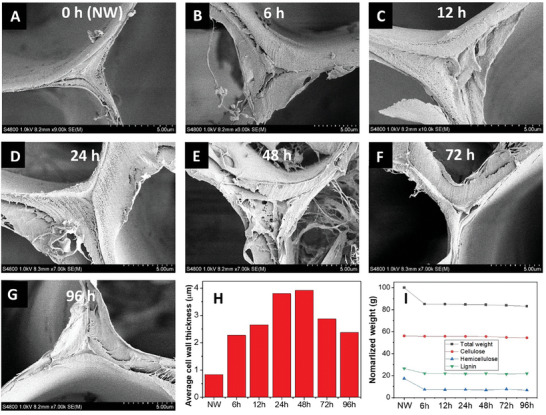
Cell wall thickness and chemical composition of swollen wood. SEM images A–G) show the cell wall thickness change of the substrates during NaOH treatment with different treatment times (from 0 h to 96 h). H) shows the average (from 100 cells) double cell wall thickness of the substrates. I) shows the wood total remaining weight and the change in weight of the main components.

The procedure in Figure 1 was successful in terms of strong cell wall swelling. The microstructures of the NaOH‐treated, washed and freeze‐dried wood substrates are presented in **Figure** **2** (A–G, NaOH treatment time from 0 to 96 h, type I samples, sample details are explained in the experimental section). Cell wall thickness is increased for all the treated wood substrates as shown in Figure 2A–G. Figure 2H presents the average double cell wall thickness (obtained from 100 cells) for all substrates. With increased treatment time, cell wall thickness first strongly increases (from ≈0.8 µm for 0 h to ≈4 µm for 48 h), then it starts to decrease (from ≈4 µm for 48 h to ≈2.5 µm for 96 h). The early increase is from NaOH‐induced swelling and the late decrease is because of cell wall material loss due to dissolution and diffusion into lumen space. Stone and Scallan observed the same phenomenon during delignification of wood fibers.^[^ ^34^ ^]^ The maximum cell wall swelling in the present study, however, is remarkably high. It is far beyond the water‐swollen state (Figure 2A) typical for wood modification and polymer‐filled wood composites. It was also confirmed that the main cell wall weight loss (≈15%) was taking place during the first 6 h of treatment (Figure 2I). There is almost no decrease in the absolute weight of cellulose. The lignin fraction is slightly decreased, while the major weight loss is hemicelluloses. Figure S1 (Supporting Information) shows that the characteristic cell structure of wood is preserved for all NaOH treatment times.

Aq. NaOH treatment of cellulose at sub‐zero temperature can be used for cellulose dissolution.^[^ ^32^ ^]^ The mechanism is related to the increased hydration of Na^+^ and OH^−^ ions at low temperature. Yamashiki et al. investigated this in detail for NaOH and LiOH^[^ ^32^ ^]^ using nuclear magnetic resonance and reasoning in terms of hydrogen bond breakage, although additional analysis of changes in the free energy of mixing are needed. The conclusion is that specific ion‐water structures are forming, which are essential for cellulose swelling. Hemicelluloses are also strongly influenced by sub‐zero aq. NaOH and their content is decreased (Figure 2I). Removal of hemicelluloses facilitates cell wall swelling since they can be strongly adsorbed to cellulose fibrils, bonding fibrils together.^[^ ^35^ ^]^

### 2.2 Cell Wall Nanostructure

**Figure 3:**
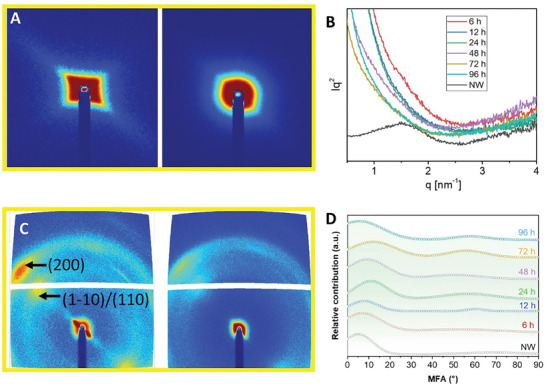
Structural information of swollen wood. A) shows the SAXS data of native wood and 96 h NaOH treated sample (other samples see Figure S2A, Supporting Information). B) SAXS Kratky plots for cellulose fibril correlation distance estimation. C) shows the WAXS data of native wood and 96 h NaOH treated wood (other samples see Figure S2B, Supporting Information), for cellulose crystal structure. D) corresponds to the microfibril angle, MFA, of wood (peak position) as a function of NaOH treatment time (curves were vertically shifted for data interpretation).

The observed cell wall thickness change is a result of nanostructural changes in the cell wall. To investigate the nanofibril aggregation/organization changes, small‐angle X‐ray scattering (SAXS) measurements were performed. **Figures** **3**A and S2A (Supporting Information) show the 2D‐SAXS patterns. The native wood shows anisotropically ordered 2D‐SAXS patterns, while after NaOH treatment, the wood substrate (type I) shows a loss of order, possibly due to increased and more irregular interfibril correlation distance. Figure 3B shows the Kratky plots, I(q)*q^2^ versus q for analysis of this structural order. Native wood shows a peak, representing a correlation length (average distance between adjacent cellulose microfibrils in the cell wall) for native balsa of ≈4 nm. This peak is absent in the treated substrates since there is a loss of order and a strong increase in interfibril distance because of the cell wall swelling. As a consequence, nanopores are formed in the cell wall, which are beneficial for the accessibility of chemical reagents (e.g., monomers) so that the wood cell wall can become a wood‐polymer nanocomposite.

The washed cellulose structures at crystallographic scale were investigated by wide‐angle X‐ray scattering (WAXS) and the results are shown in Figure 3C and Figure S2B (Supporting Information). After NaOH treatment, the intensity of WAXS patterns is reduced (Figure 3C), possibly an effect of lowered cell wall density. Native balsa obviously shows typical cellulose (1‐10)/(110) and (200) characteristics. With increasing NaOH treatment time, there are no apparent changes in the cellulose crystal type of the washed samples. The question is why there is no transition from cellulose I to cellulose II. Pure cellulose samples with negligible hemicellulose content can form cellulose II under similar conditions as used here.^[^ ^36^ ^]^ In our wood samples, the presence of lignin and hemicelluloses, the lack of agitation, and the thickness of the cell wall (µm‐scale) are factors that decrease the efficiency of dissolution. Hydrated Na^+^ and OH^−^ ions are associated with hemicelluloses and lignin, instead of cellulose, decreasing the effective NaOH concentration. With agitation of a cellulosic particle suspension, a fresh solvent is brought to cellulose crystal surfaces, whereas with the present solid wood substrate, this is not the case. The phrase “the presence of lignin and hemicelluloses” means that fibrils cannot merge and form antiparallel molecular conformation, which is a requirement for cellulose II formation.^[^ ^37^ ^]^ The main objective, strong cell wall swelling, is achieved and there are no detrimental changes to the cellulose I structure.

Cellulose crystal size (Scherrer size) of the cellulose (200) reflection is decreased for the NaOH‐treated samples as shown in Table S1 (Supporting Information), and is associated with reduced cellulose crystallinity (long‐range order, **Table** **1**). Most likely, the reason is decreased order in the cellulose fibrils due to the formation of swollen Na‐cellulose I, followed by rinsing and removal of sodium ions.^[^ ^37^ ^]^ Microfibril angle (MFA) shows an increase with increased treatment time as is also illustrated in Figure 3D (source: Figure S3, Supporting Information). Native wood shows an average MFA ≈5°, while it increases (up to 12°) with increased NaOH treatment time. Increased MFA correlates with ≈10% axial shrinkage of the wood substrate after aq. NaOH treatment and washing. At 96 h treatment time, the MFA is lower for unknown reason, possibly due to the low initial MFA of the specific sample.

**Table 1:** Cellulose crystallinity (estimated based on the method by Segal^[^ ^38^ ^]^) and specific surface area for wood substrates with different NaOH treatment times.

NaOH treatment time [h]	Crystallinity [%]	Specific surface area [m^2^ g^−1^]
0 (NW)	71.4	8
6	68.8	16
12	63.5	65
24	63.6	204
48	62.9	163
72	62.6	150
96	61.6	180

**Figure 4:**
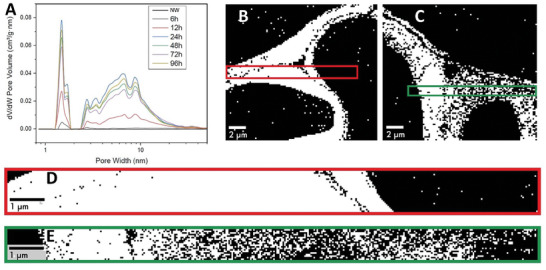
Structural information of swollen wood. A) shows the nanoporosity of the samples during the treatment according to BET data. B) and C) show processed images based on scanning electron diffraction data of the cell wall of PMMA‐filled native wood and NaOH‐treated wood (96 h), respectively. White represents areas with characteristic diffraction from cellulose, and black represents areas without cellulose. D) and E) are the magnified cell wall region from B) and C), respectively.

Due to the cell wall swelling from sub‐zero aq. NaOH treatment, nanoporosity of the cell wall increases according to BET data, see **Figure** **4**A. More pores are formed with a diameter below 10 nm, in agreement with lignin removal effects during pulping.^[^ ^34^ ^]^ This is in support of the SAXS data in Figure 3B. The specific surface area (SSA) for the treated wood substrates is also increased (see Table 1). For native balsa, SSA is 8 m^2 ^g^−1^, while NaOH‐treated wood (24 h) reaches 204 m^2 ^g^−1^. This is very favorable for wood modification purposes. The increase is a combined effect of cell wall swelling and weight loss of wood components (mainly hemicellulose) as shown in Figure 2I. After 24 h treatment, SSA starts to decrease (down to 150 m^2^ g^−1^ for 72 h). The reason is that some of the high SSA wood components diffuse from the cell wall into the empty, central lumen space. The substance in the lumen is clearly visible in the micrographs in Figure S1 (Supporting Information) for 48, 72, and 96 h treatment, and the more disorganized structure of components in the lumen is likely to decrease SSA. It is not apparent why SSA is higher (180 m^2^ g^−1^) for 96 h treatment, but this sample also showed a typical MFA.

The two substrates used for SAXS characterization (native and NaOH‐treated) were afterward impregnated by MMA which was polymerized to form two different wood/PMMA composite samples intended for additional analysis. The nanostructures of the cell walls in the two composites were then characterized by scanning electron diffraction (SED) which is suitable to analyze the organization of cellulose in hybrid materials,^[^ ^39^ ^]^ see Figure 4B,C (the regions are from the STEM images in Figure S4, Supporting Information). The white regions represent domains with characteristic diffraction from cellulose; the dark regions represent amorphous domains without cellulose. The cell wall in Figure 4B (native wood/PMMA) is almost completely white and dominated by cellulose (see also enlarged image in Figure 4D). The NaOH‐treated wood/PMMA composite (Figure 4C) shows more dark regions without cellulose in the cell wall (see also the image in Figure 4E). During preparation of this composite, MMA monomer diffused into the highly porous and swollen cell wall so that the cell wall itself became a nanostructured composite. Higher cell wall swelling will increase small‐scale cell wall porosity which facilitates cell wall impregnation by MMA. The presence of PMMA in the cell wall is supported by Figure S4 (Supporting Information) (no cell wall pores) and was confirmed in a previous TW study where we used small‐angle neutron scattering and SAXS.^[^ ^15^ ^]^

### 2.3 Mechanical Properties

**Figure 5:**
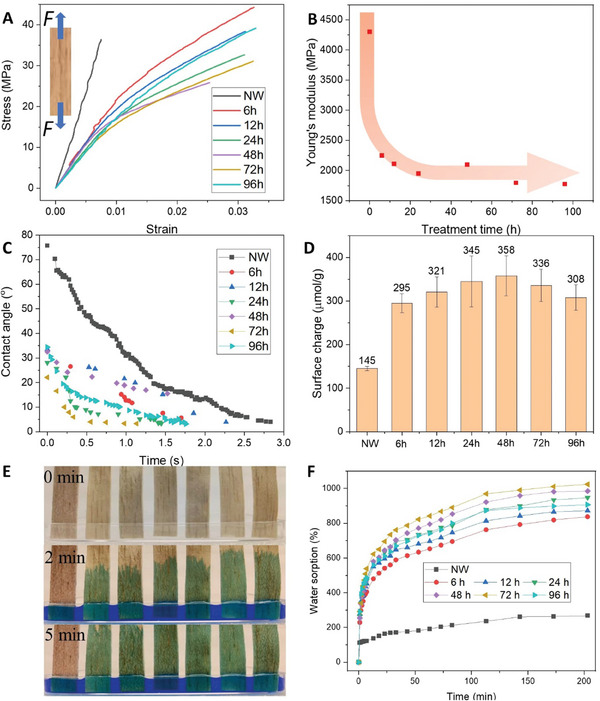
Properties of NaOH‐treated wood substrates as a function of treatment time. NW is untreated native wood. A) stress‐strain curves from axial tensile tests, and B) axial Young's modulus, C) water contact angle of wood substrate versus time and D) total surface charge versus treatment time. E) illustrates dyed water uptake with time for various NaOH treatment times from 0 h (most left) to 96 h (most right) (top: 0 min, middle: 2 min, bottom: 5 min). F) water sorption versus time.

To investigate the influence of NaOH treatment on the mechanical behavior of the low‐density wood substrates, stress‐strain measurements (type II samples) for uniaxial loading in axial direction were performed, and the curves are presented in **Figure** **5**A. The stiffest and strongest material has been treated in the shortest time (6 h). For longer treatment times, the wood substrate becomes more ductile than for 6 h treatment and the shape of the stress‐strain curve becomes more non‐linear. Strain to failure increases substantially (≈3.3%) compared to native wood (≈0.7%) and strength is reduced at longer treatment times. The 96 h samples do not follow the trend of reduced properties with longer treatment time. Figure 5B presents the axial Young's modulus of the substrates, where all the treated substrates show less than half the value (≈1770 MPa after 96 h) of native balsa (≈4300 MPa). Note that the specimens are freeze‐dried with substantial cell wall porosity, the MFA has increased (Figure 3D) and 15% of the cell wall components have been removed (hemicelluloses and some lignin). The load transfer between cellulose fibrils (main load‐bearing component in cell wall) is reduced when some of the lignin/hemicellulose “glue” between fibrils is removed and by increased cell wall porosity. Higher MFA also reduces axial modulus.

### 2.4 Surface Property and Water Uptake

The surface properties of the substrates are also influenced by the treatment. As shown in Figure 5C, the substrate (type I samples) surfaces show lower water contact angle and increased hydrophilicity compared to native wood. Figure 5D shows that total electronegative charge of the treated substrates (type I) is more than twice as high (up to 358 µmol g^−1^) as for native wood (145 µmol g^−1^). The main reason is probably increased carboxylates from hemicelluloses, and phenoxides in lignin after NaOH treatment.^[^ ^33^ ^]^

The increased cell wall nanoporosity and SSA increase the accessibility of the NaOH‐treated wood cell walls. They contain nanoscale cellulose fibrils but have reduced hemicellulose (−60%) and lignin content (−20%). To further investigate the effects of this, samples (type II) treated for different times (from 0 to 96 h) were positioned in a Petri dish as shown in Figure 5E (top for time at 0). Dyed water was added and all samples rapidly sorbed water from the bottom, by capillary effects. After 2 min, see Figure 5E (middle), it is apparent that longer NaOH treatment times increase the rate of water sorption. After 5 min, in Figure 5E (bottom), all treated samples are filled with water, while water sorption in native wood (0 h) is more limited and not apparent (also see supporting video). Water sorption is reported as a function of time in Figure 5F. NaOH‐treated samples (type I) sorb more water than native wood. The value is up to 10 times the initial dry weight (72 h), which is around five times the weight increase for native wood. The main reason is strongly increased capillary effects from lignin/hemicellulose removal (facilitates swelling), increased nanoporosity, SSA, surface charge, and hydrophilicity.

### 2.5 Large‐Scale Wood Delignification and Transparent Wood Fabrication

**Figure 6:**
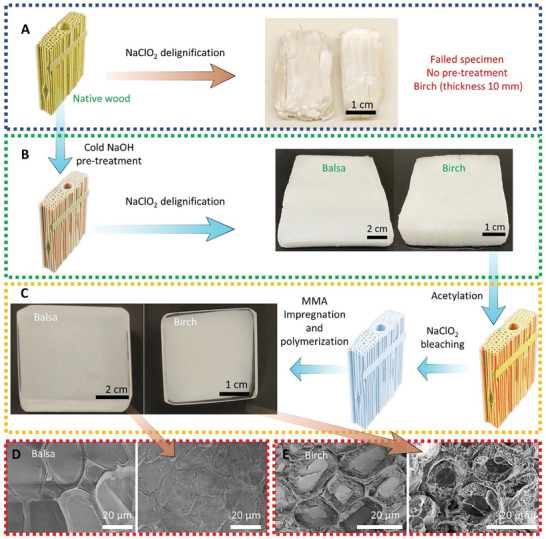
Fabrication of transparent wood composites. A) Native birch subjected to delignification without pretreatment. B) successfully delignified wood substrates of large size subjected to sub‐zero temperature NaOH pretreatment. C) schematic of thick transparent wood sample preparation. D) SEM images of acetylated balsa‐based TW without (left) and with (right) NaOH pretreatment. E) SEM images of acetylated birch‐based TW without (left) and with (right) NaOH pretreatment.

To validate the increased accessibility of the NaOH‐pretreated wood substrate, NaClO_2_‐based delignification was performed on samples. Native birch (with a thickness 10 mm) was subjected to NaClO_2_ treatment. As shown in **Figure** **6**A, these birch substrates disintegrated after chemical treatment due to gradients in the concentration of NaClO_2_ where the interior of the sample was not readily accessible. Fibers were disintegrated in the surface region, whereas the interior was incompletely delignified. In contrast, Figure 6B shows thick samples that were successfully delignified: balsa (type III sample, 15 mm thickness) and birch (type IV sample, 10 mm thickness) after NaOH pretreatment. We have not found reports of successful delignification of such large‐sized wood substrates with fibers parallel to the wood surface. The delignified wood substrates were then acetylated, bleached, and impregnated with MMA to prepare TW composites as shown in Figure 6C, based on our previous methods.^[^ ^40^ ^]^ SEM micrographs for both balsa (Figure 6D) and birch‐based TW (Figure 6E) show that wood cell walls are in thick, swollen state also in TW composite cross‐sections, due to the NaOH‐treatment.

### 2.6 Optical Properties of Transparent Wood

**Figure 7:**
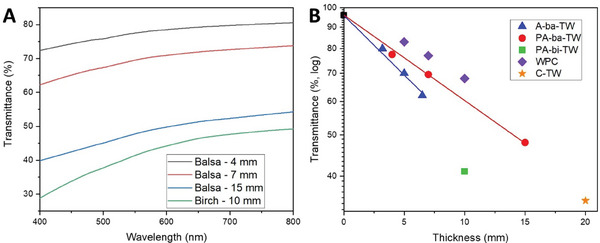
Transparent wood optical properties. A) Optical transmittance of TWs with various thicknesses. B) Transmittance at a wavelength of 550 nm and sample thicknesses from literature and data from the current study. A‐ba‐TW is data for PMMA/acetylated balsa,^[^ ^42^ ^]^ PA‐ba‐TW is present data for sub‐zero NaOH pretreated PMMA/acetylated balsa (≈10 vol % wood), PA‐bi‐TW is for sub‐zero NaOH pretreated PMMA/acetylated birch (≈25 vol % wood), WPC is for wood flour/PMMA,^[^ ^9^ ^]^ and C‐TW is for sample with fibers perpendicular to the surface.^[^ ^43^ ^]^

In **Figure** **7**A, optical transmittance for balsa‐based TW of different thicknesses (4, 7, and 15 mm, respectively) and birch‐based TW (thickness 10 mm) are presented. Transmittance decreases with increased thickness, as expected due to the increased number of interfacial scattering sites (PMMA‐wood cell wall).^[^ ^41^ ^]^ To compare the present optical transmittance to previous studies, optical transmittance (at wavelength 550 nm) and sample thickness data are summarized in Figure 7B. The data are used to quantify the thickness dependence of transmittance. The well‐known Beer‐Lambert Law is strictly only valid for non‐scattering materials. For anisotropic materials with scattering, such as TW, we use a model based on photon diffusion. The model for total transmittance *T_tot_* has the same form as the Beer‐Lambert Law

(1) $$T_{tot}=e^{-\alpha d}$$

where *d* is sample thickness, but the attenuation coefficient *α* is completely different in nature since it depends on two anisotropic diffusion coefficients in addition to a coefficient for light absorption.^[^ ^42^ ^]^ The attenuation coefficient *α* is the key measure of optical transmittance. For NaOH pretreated and acetylated balsa‐based TW (PA‐ba‐TW) the present attenuation coefficient *α* is 0.48 cm^−1^ at a wavelength of 550 nm (obtained through the fitting of the red circles in Figure 7B). This is an improvement compared with the previous result (*α* = 0.64 cm^−1^) for acetylated TW without NaOH pretreatment (A‐ba‐TW), obtained through the fitting of blue triangles in Figure 7B).^[^ ^42^ ^]^ This is due to increased polymer content in the cell wall. A more homogeneous mixture of cell wall nanofibrils and polymer phase in the cell wall contributes to decreased refractive index mismatch between polymer in the lumen and the cell wall nanocomposite (as shown in Figure 6D) and possibly fewer interface defects. In addition, NaOH pretreated and acetylated birch‐based TW (PA‐bi‐TW) was prepared with a thickness of 10 mm (morphology comparison between birch TWs with and without pretreatment is shown in Figure 6E), the transmittance at 550 nm is reported in Figure 7B (green square). The transmittance of the high wood content (≈25 vol %) birch TW reported here (green square) was roughly similar and comparable to acetylated balsa‐based (≈10 vol % wood) TW (blue triangles). This is a substantial improvement in thick TW of high wood content in terms of optical transmittance.

Figure 7B also contains data for a wood flour/polymer composite (WPC, 10% wood particles).^[^ ^9^ ^]^ The data show higher optical transmittance than for TW. This material contains low aspect ratio wood particles and has low strength since fiber strength is not important for fracture. If TW is designed with a fiber direction perpendicular to TW surface (out‐of‐plane), the transmittance is also improved^[^ ^43^ ^]^ (C‐TW in Figure 7B, 35% transmittance at 20 mm thickness). Again, a major drawback is the very low in‐plane tensile strength (≈21 MPa) since the high tensile strength in the fiber direction cannot be utilized. In previous publications, most reports were on TW sample thicknesses 3 mm or lower. The small thicknesses and differences between different studies (often data are only for a single thickness), see Figure S5 (Supporting Information), means comparisons with previous data are not meaningful for the present range of large thicknesses. Haze data for balsa‐based TW with various thicknesses (4, 7, and 15 mm, respectively) and birch‐based TW with the thickness of 10 mm are presented in Figure S6 (Supporting Information). Haze values are higher than 90%, so there is plenty of room for reduced TW light scattering.

## 3 Conclusion

Increased cell wall accessibility is of critical importance to wood nanotechnologies. Immersion of wood in green NaOH solution at sub‐zero centigrade temperature resulted in extensive cell wall swelling beyond the native water‐swollen state of wood. The major swelling occurred inward in each cell so that the central empty space was decreased; increased hydration of ions from NaOH at sub‐zero temperature was important.

The sub‐zero treatment resulted in a doubling in total electronegative charge; unique cell wall swelling effects resulted in a large increase of total cell wall porosity, increases in the fraction of nanoscale size pores, specific surface area (≈200 m^2^ g^−1^) and distance between oriented cellulose fibrils in the cell wall. With this pretreatment, thick wood structures (≈15 mm) could be readily delignified without large through‐thickness gradients in composition.

Compared with nanostructural tailoring possibilities in typical polymer nanocomposites, this procedure has advantages since the oriented nanofibril organization of the wood cell wall is preserved in composites, including polymer matrix well distributed at nanoscale. Transparent wood composites of large thickness and improved optical transmittance were prepared. Bring the polymer matrix into the cell wall was facilitated by the NaOH‐induced swelling. The refractive index mismatch between cell wall and the polymer phase inside fibers was reduced due to the nanocomposite nature of the cell wall. Sub‐zero NaOH pretreatment of wood combines large swelling effects of the cell wall with preserved cellulose I structure for mechanical performance. In future work, swelling mechanisms at several length‐scales (molecular, mesoscale and cell wall microscale) need clarification.

## 4 Experimental Section

### Materials and chemicals

Balsa wood (density 90 kg m^−3^, purchased from Materials AB, Sweden) with dimension 10 × 10 × 10 mm (type I), 50 (wood fiber direction) × 10 × 1 mm (thickness direction perpendicular to the wood fiber direction) (type II), 100 × 100 × 15 mm (type III, 140 kg m^−3^), and birch wood (type IV, 620 kg m^−3^, purchased from Materials AB, Sweden) with dimension 45 × 45 × 10 mm, respectively, were prepared (immersed in water for 10 days) for Sodium hydroxide (NaOH) treatment. NaOH was purchased from Sigma Aldrich, Sweden.

### Sub‐zero NaOH Solution Treatment and Delignification

Pre‐cooled water‐soaked balsa wood (4 °C) was introduced to the pre‐cooled (−6 °C) NaOH/water solution (7 wt. %) and then kept at −6 °C for 6, 12, 24, 48, 72, and 96 h for type I and type II samples, respectively. For type III and type IV samples, the treatment time are 30 and 45 h, respectively. The volume ratio of wood: NaOH solution is 1: 50. All the NaOH‐treated substrates were washed with deionized water until neutral pH. The treatment process is shown in Figure 1. Finally, type III and type IV wood substrates were delignified using 1 wt. % of Sodium Chlorite (NaClO_2_, Sigma–Aldrich) with acetate buffer solution (pH 4.6) at 80 °C until the wood template changed to white.

### Morphology and cell wall Thickness Measurement

The cross‐section of the wood substrates (type I) was obtained by microtome and then freeze‐dried. The sample morphology was characterized by scanning electron microscopy (FE‐SEM, Hitachi S‐4800, and tabletop SEM‐TM1000, Japan). The double cell wall thickness is measured using Image J software, based on the obtained SEM images of the cross‐section of the wood substrates. The average number of measured cells is 100.

### Chemical Analysis

Carbohydrate and lignin contents were measured. First, the wood substrate (type I) was milled to powders using a Wiley mill. Then the oven‐dried (105 °C) powder (200 mg) was hydrolyzed in sulphuric acid (73 wt. %) and autoclaved to complete hydrolysis. Klason lignin content was determined according to TAPPI T 222 om‐2.^[^ ^44^ ^]^ The monosaccharide content of the acid‐soluble part was analyzed using a chromatography system (Dionex ICS‐300 ion chromatography, Thermo Fisher Scientific Inc., USA).

### Structural Information

Structural information (type I samples) at the nanoscale (≈0.3–13 nm) and microfibril angle (MFA) were investigated by using small‐angle X‐ray scattering (SAXS) and wide‐angle X‐ray scattering (WAXS). SAXS and WAXS measurements were performed on a point‐collimated X‐ray system (Anton Paar, Cu Kα radiation,) with a beam size of 500 µm, wavelength 1.5418 Å, and an Eiger R 1 M detector with 75 × 75 µm pixel size. All measurements were carried out at room temperature under vacuum conditions. The sample‐to‐detector distance was set to 576 mm and 111 mm for SAXS and WAXS, respectively. WAXS measurements for MFA calculation were performed with a Xenocs Xeuss 3.0, a Cu K alpha X‐Ray source (GeniX 3D), and a Dectris EIGER2 1 M detector placed at a sample detector position of 50 mm (distance calibrated using a LaB_6_ standard). To have a continuous q‐range from 0.5 Å^−1^ to 3.5 Å^−1^, the line eraser mode was used, which collects two images (150 s exposure per image) at slightly altered detector positions to allow a seamless q‐range. The azimuthal profile was obtained by integrating around the q‐crown between 1.4 and 1.75 Å^−1^. The MFA distribution was determined using the previously reported protocol and software by Rüggeberg et al.^[^ ^45^ ^]^ All data handling was performed using the Xenocs XSACT software. In all scattering measurements, the wood substrates were mounted with the beam perpendicular to the longitudinal fiber direction of the wood. The 1D curves with Intensity (I) versus scattering vector (q) were obtained by integrating the whole region of 2D images. The crystallinity was calculated based on Segal's method.^[^ ^38^ ^]^

### Specific surface Area (SSA) and Pore Size Distribution (PSD)

All the samples (type I) were first dried using a CO_2_ critical point drying (CPD, Autosamdri‐815), then heat treated in dynamic vacuum (1 × 10^−4^ Pa) at 105 °C for 24 h using a Micromeritics SmartVacPrep 060 (USA). The SSA and PSD of the sample were analyzed using nitrogen sorption isotherms collected using a Micromeritics ASAP2020 gas adsorption analyzer (USA). SSA was calculated following by Brunauer‐Emmett‐Teller (BET) theory^[^ ^46^ ^]^ for p/p_0_ ≈0.05–0.20. PSD was estimated using the Density Functional Theory (DFT) function in the Micromeritics MicroActive software (slit pore model).

### Scanning Electron Diffraction (SED)

Ultra‐thin sectioning of the two samples (type I, native balsa and NaOH treated balsa for 96 h, solvent exchanged from water to acetone, infiltrated with MMA and then polymerized), with an estimated section thickness of 200 nm, was conducted using a Leica Ultracut UCT at room temperature with a 35° diamond knife from Diatome. The resulting sections were later transferred onto carbon‐coated copper grids (EMS‐CF150‐Cu‐UL). Characterization was performed using a double aberration‐corrected Thermo Fisher Themis‐Z transmission electron microscope operating at 300 kV, equipped with a CheeTah M3 hybrid electron camera (512 × 512 pixels) from Amsterdam Scientific Instruments. The microscope was configured to microbeam mode with a camera length of 360 mm and a beam convergence angle of 0.1 mrad, resulting in a nearly parallel beam with a diameter of less than 10 nm. Raster scanning was controlled via the Gatan Microscopy Suite using a beam current of 2 pA to minimize beam‐induced damage. For STEM imaging, the dwell time was set to 16 µs, while for SED recordings, it was set to 5 ms. Data processing involved a custom‐made Python script utilizing a combination of open‐source libraries such as Numpy, Hyperspy, and SciPy. This script positioned 360 virtual detectors in a ring around the direct beam, with a radius corresponding to the scattering angle of the cellulose 200 reflections. Next, the two semicircles (1–180 and 181–360 degrees) were added to enhance the signal‐to‐noise ratio, as Bragg diffraction is symmetrical. A threshold value based on the standard deviation was then used to differentiate cellulose diffraction from random scattering.

### Tensile Tests

The sample (type II) tensile tests were performed using an Instron 5944 equipped with a 500 N load cell and a video extensometer. Young's modulus is calculated by linear fitting of the initial elastic region of the stress‐strain curves. Before the test, samples are preconditioned for 48 h in a room with controlled temperature (22 °C) and relative humidity (50%).

### Contact Angle

The contact angle of the sample (type I) surfaces was measured using an Optical Tensiometer (Theta Lite, Biolin Scientific, Finland).

### Total Charge

The total amount of electronegative groups in the wood samples (type I) was determined using conductometric titration (Metrohm 702 SM Titrino titrator, Switzerland).

### Water Uptake

For water uptake rate, freeze‐dried wood samples (type II) obtained from different NaOH treatment times (from 0 to 96 h) were placed in a Petri dish, then the blue dye water was added to the dish. The water uptake process is recorded by a camera (supporting video). The water uptake was measured by immersing the freeze‐dried samples (type I) into deionized water, then all the samples were weighed. The time interval for each measurement is 5 min. Finally, the water uptake is calculated by:

(2) $$Water\;\;uptake\;\;capacity\;\;\left(\%\right)=\frac{m_{2}}{m_{1}}\;\times 100\%$$

where m_1_ is the initial dry wood weight, and m_2_ is the absorbed water weight.

### Large‐Scale Transparent Wood Preparation

The successfully delignified balsa (type III) and birch wood substrates (type IV) were used for transparent wood fabrication according to the previous method.^[^ ^40^ ^]^ First, the delignified wood substrates are solvent exchanged from water to ethanol, acetone, and finally N‐methyl‐2‐pyrrolidone (NMP, Sigma‐Aldrich), respectively (each process repeated at least 3 times). Second, wood acetylation was performed using acetic anhydride (Sigma‐Aldrich) with pyridine (Sigma–Aldrich) as the catalyst and NMP as the solvent. The volume ratio of acetic anhydride:pyridine:NMP is 7:6:100. Due to the large thickness of the wood substrate, acetylation was conducted for 10 h. Third, wood substrates were solvent exchanged from the reaction chemical to acetone, ethanol, and finally, water (each process repeated at least 3 times) to remove the residual chemicals. After that, wood substrates were bleached until white to remove the brown color resulting from acetylation. Then the white wood substrates were solvent exchanged from water to ethanol and acetone, respectively (each process repeated at least 3 times), and finally impregnated with PMMA under vacuum for 5 days to ensure the complete infiltration of PMMA. The packaged wood substrates with PMMA were finally polymerized in the oven, with the gradually increased temperature from 30 to 70 °C.

### Optical Transmittance and Haze

The angle‐integrated total optical transmittance and haze of TWs were measured with an integrating sphere according to ASTM D1003 “Standard Test Method for Haze and Luminous Transmittance of Transparent Plastics.”^[^ ^47^ ^]^

## Conflict of Interest

The authors declare no conflict of interest.

## Supporting information



Supporting Information

Supplemental Video 1

## Data Availability

The data that support the findings of this study are available from the corresponding author upon reasonable request.
