# Supplementary material for: Green Nanotechnology of Cell Wall Swelling for Nanostructured Transparent Wood of High Optical Performance
Source: Small. 2024 Dec 17;21(5):2406749. doi: 10.1002/smll.202406749 (PMC11798347; doi:10.1002/smll.202406749)
Supplement: Supplementary file 1 — Supporting Information [file SMLL-21-2406749-s001.docx]

Supporting Information

**Green nanotechnology of cell wall swelling for nanostructured transparent wood of high optical performance**

*Hui Chen, Jonas Garemark, Lengwan Li, Mathias Nero, Maximilian Ritter, Ocean Cheung, Tom Willhammar, Ilya Sychugov, Yuanyuan Li*, and Lars A. Berglund**

*Email: yua@kth.se, blund@kth.se


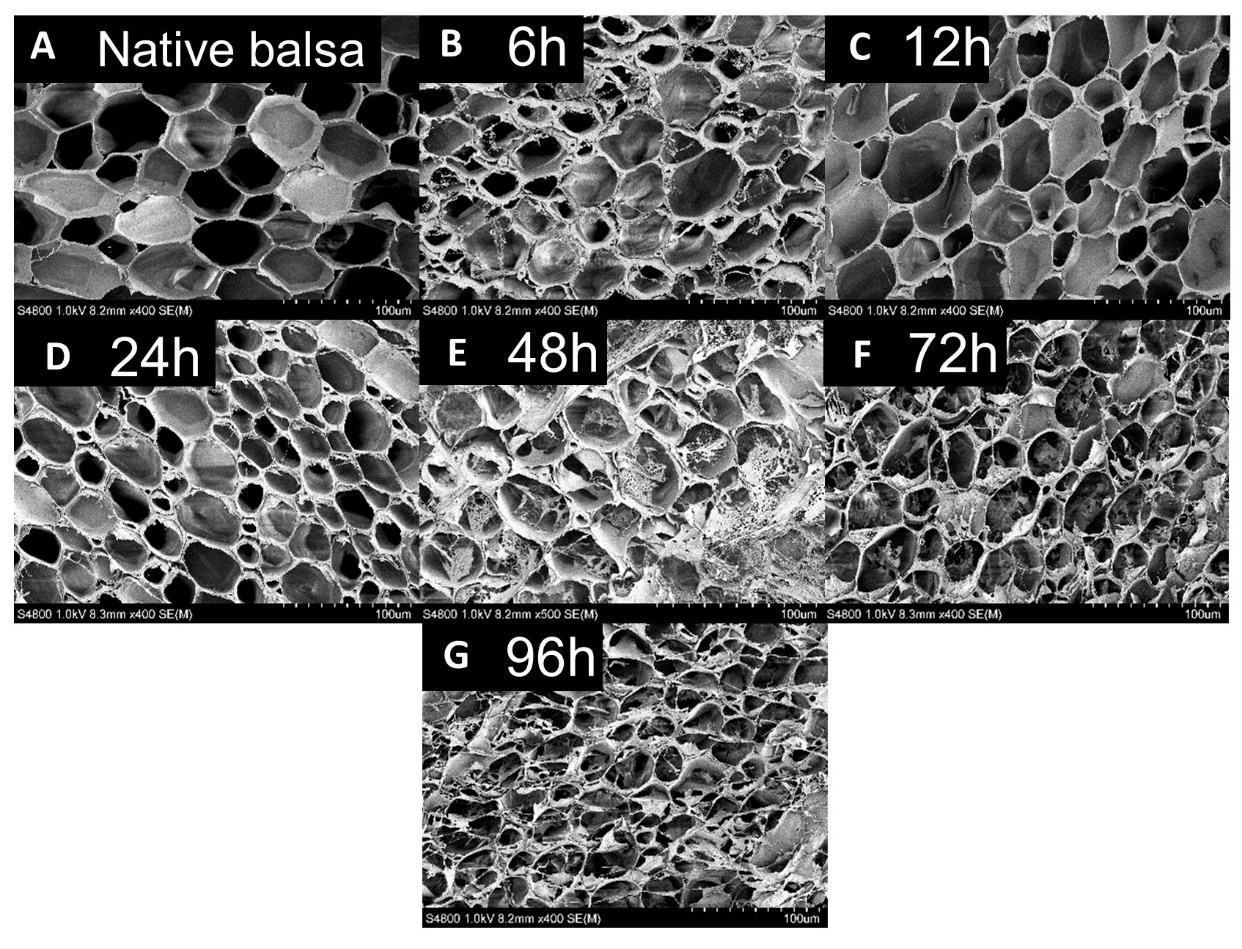


**Figure S1**. SEM images A-G show the preserved wood structure of the substrates during NaOH treatment with different treatment times (from 0 h to 96 h).


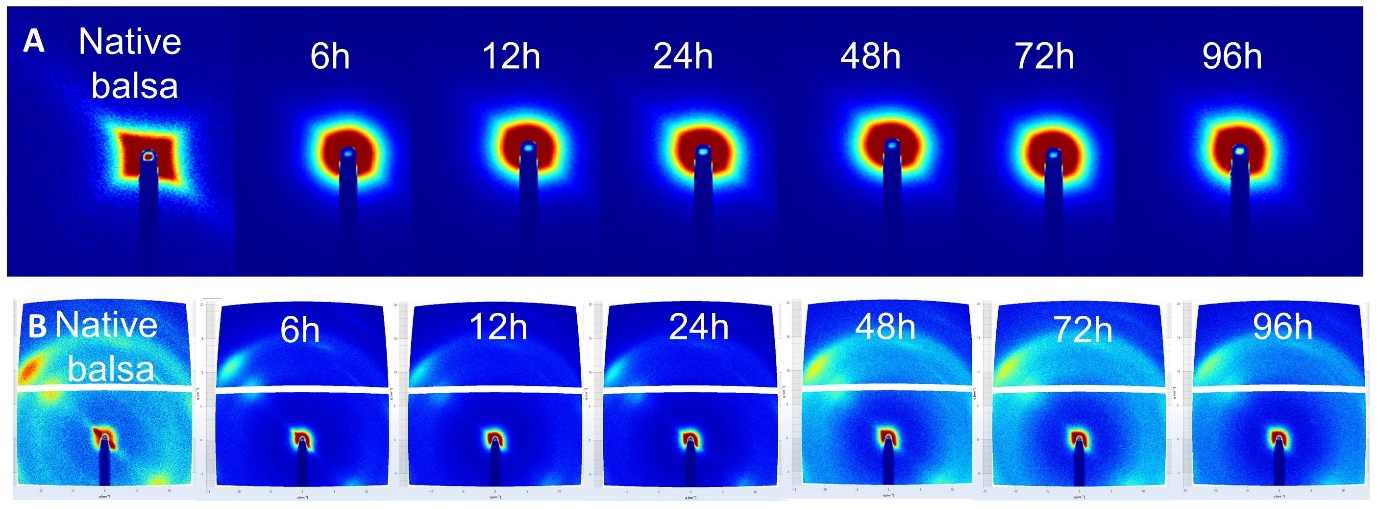


**Figure S2.** A) SAXS and B) WAXS of balsa wood treated with cold NaOH with different times (from 0 h to 96 h).


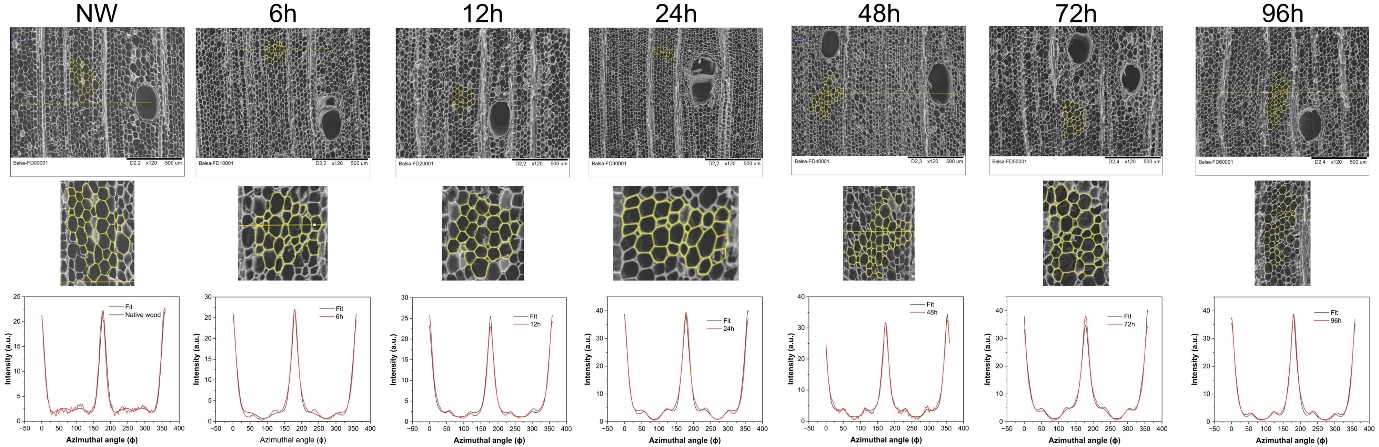


**Figure S3.** The top and middle images represent cross-sections of the samples (NW, 6h, 12h, 24h, 48h, 72, and 96h); straight yellow lines indicate the direction of the X-ray beam, and yellow honeycombs indicate cell walls to obtain length l and orientation α for MFA calculation. The last row shows the azimuthal intensity profiles after radial integration, including the fit.

**Table S1.** Scherrer size obtained from cellulose (200) reflection of the balsa samples with different NaOH treatment time, obtained by using Gaussian function according to previous method.^[1]^

| Samples | Scherrer size (nm) |
| --- | --- |
| Native | 2.18 |
| 6h | 1.77 |
| 12h | 1.38 |
| 24h | 1.34 |
| 48h | 1.38 |
| 72h | 1.34 |
| 96h | 1.36 |


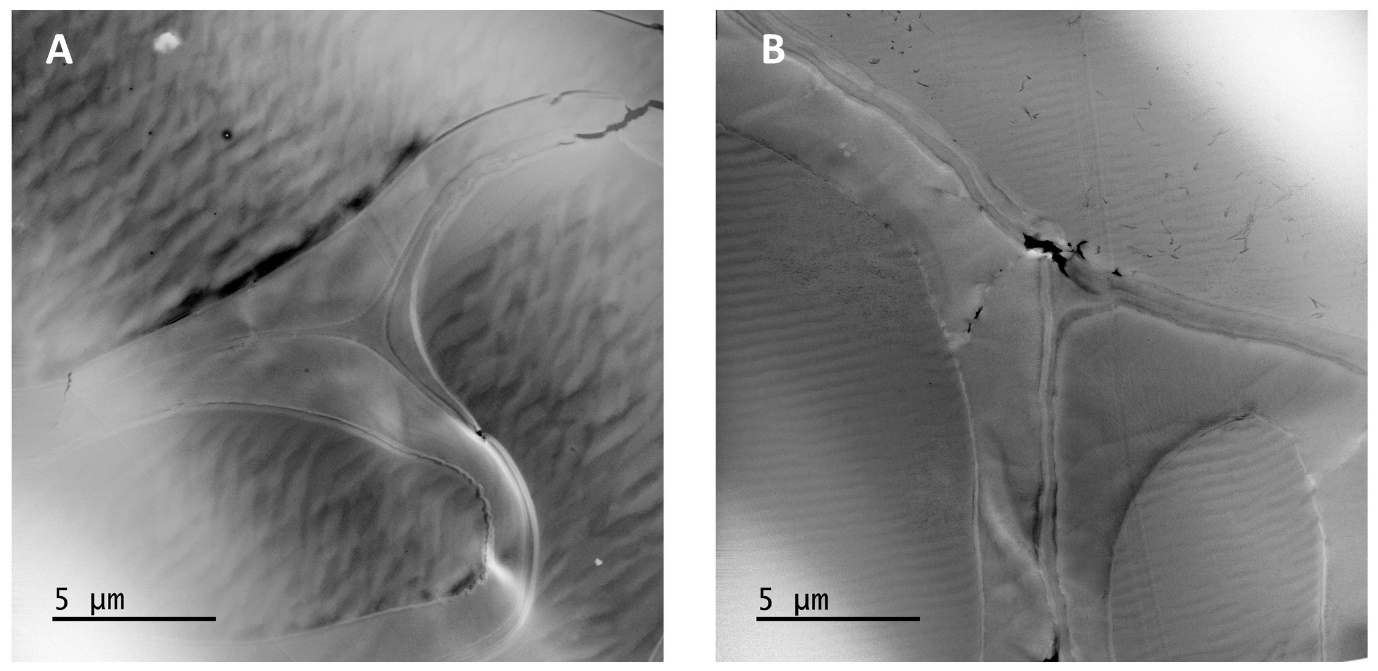


**Figure S4.** STEM image of A) native balsa wood and B) cold NaOH treated (96 h) balsa wood, both impregnated with PMMA.


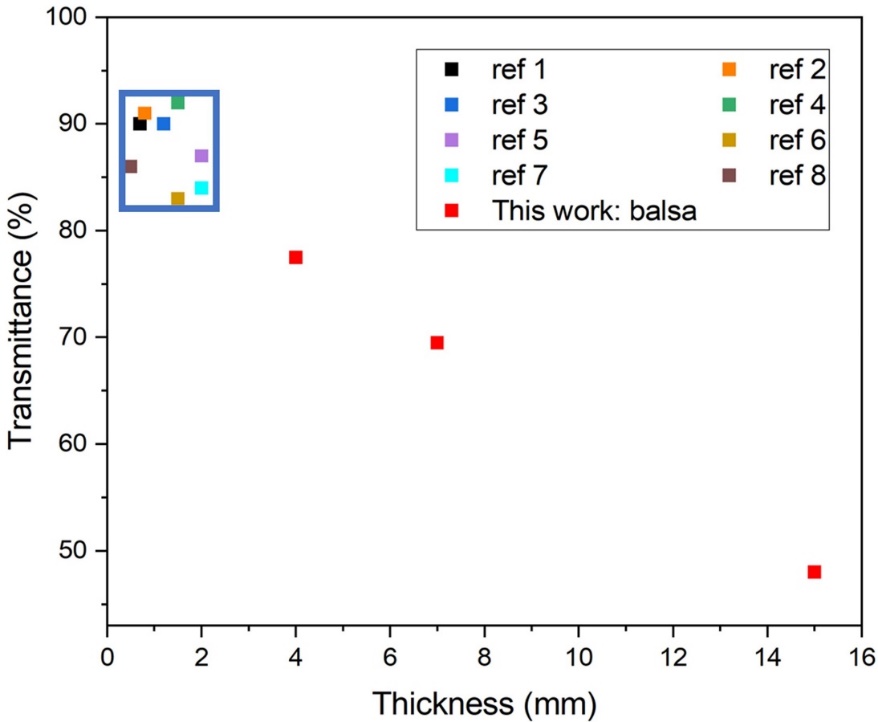


**Figure S5.** Transmittance of transparent wood with low and different thicknesses in different studies^[2–9]^ (in blue rectangle) and balsa from this work with large thicknesses (red dots).


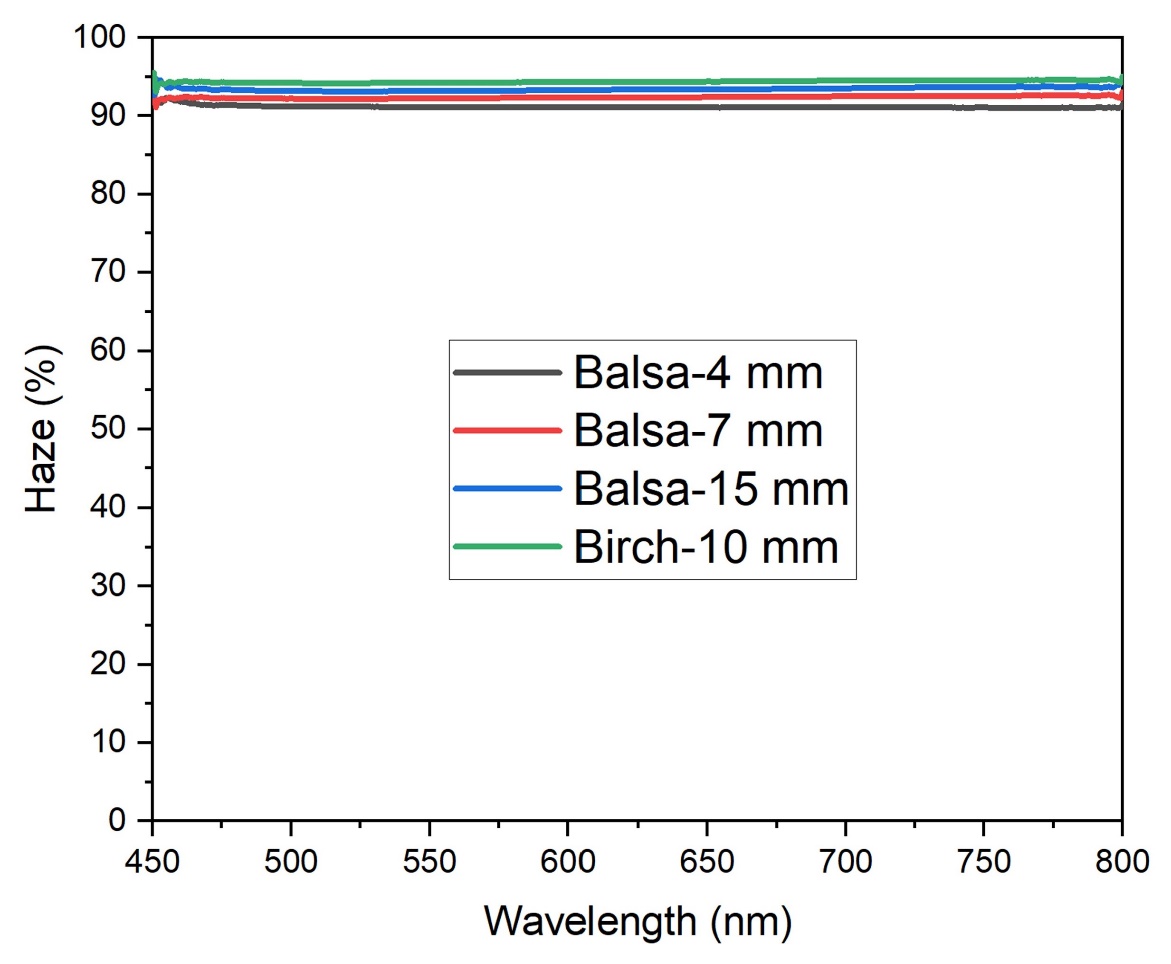


**Figure S6.** Haze of balsa-based transparent wood with thicknesses of 4, 7, and 15 mm, respectively, and birch-based transparent wood with thickness of 10 mm.

**References**

[1] L. Li, P. Chen, L. Medina, L. Yang, Y. Nishiyama, L. A. Berglund, *ACS Nano* **2023**, *17*, 15810.

[2] Y. Li, Q. Fu, S. Yu, M. Yan, L. Berglund, *Biomacromolecules* **2016**, *17*, 1358.

[3] R. Mi, T. Li, D. Dalgo, C. Chen, Y. Kuang, S. He, X. Zhao, W. Xie, W. Gan, J. Zhu, J. Srebric, R. Yang, L. Hu, *Adv. Funct. Mater.* **2020**, *30*, 1907511.

[4] M. Höglund, M. Johansson, I. Sychugov, L. A. Berglund, *ACS Appl. Mater. Interfaces* **2020**, *12*, 46914.

[5] Y. Li, X. Yang, Q. Fu, R. Rojas, M. Yan, L. A. Berglund, *J. Mater. Chem. A* **2018**, *6*, 1094.

[6] C. Montanari, Y. Ogawa, P. Olsén, L. A. Berglund, *Adv. Sci.* **2021**, *8*, 2100559.

[7] Y. Li, Q. Fu, R. Rojas, M. Yan, M. Lawoko, L. Berglund, *ChemSusChem* **2017**, *10*, 3445.

[8] C. Montanari, P. Olsén, L. A. Berglund, *Green Chem.* **2020**, *22*, 8012.

[9] W. Gan, S. Xiao, L. Gao, R. Gao, J. Li, X. Zhan, *ACS Sustain. Chem. Eng.* **2017**, *5*, 3855.
